# Light-Driven Iodine Loss and Photoluminescence Homogenization in Mixed-Halide Perovskite Semiconductors

**DOI:** 10.1021/acs.jpcc.6c02043

**Published:** 2026-07-08

**Authors:** Maya J. Lebowitz, Annie Gomez, Benjamin L. Cotts, Samuel D. Stranks, Alberto Salleo, Rebecca A. Belisle

**Affiliations:** † Chemistry Department, 8456 Wellesley College, Wellesley, Massachusetts 02481, United States; ‡ Department of Physics and Astronomy, Wellesley College, Wellesley, Massachusetts 02481, United States; § Department of Materials Science and Engineering, 6429 Stanford University, Stanford, California 94305, United States; ∥ Department of Chemistry and Biochemistry, 5457 Middlebury College, Middlebury, Vermont 05753, United States; ⊥ Department of Chemical Engineering and Biotechnology, 2152 University of Cambridge, Cambridge CB3 0AS, U.K.

## Abstract

Carrier-induced instabilities in lead halide perovskites are often investigated as either transient phenomena, e.g., photoinduced halide segregation or permanent performance changes, e.g., photodegradation, while the mechanistic links between them remain unclear. Here, we aim to connect these observations by studying a model mixed-halide system, MAPb­(Br_*x*_I_1–*x*_)_3_. By combining grazing-incidence X-ray diffraction, photothermal deflection spectroscopy, and photoluminescence measurements with hyperspectral microscopy, we investigate the role of mobile halide defects and local chemistry on reversible and long-term instabilities in these materials. Our results show that mixed-halide perovskites are uniquely susceptible to photoinduced changes with illumination driving initial iodide redistribution (i.e., halide segregation), eventual selective iodine expulsion, and subsequent changes in photoinduced halide segregation behavior. By quantifying structural and compositional changes, we estimate an approximately 3–5% iodine loss in our mixed-halide samples after only 24 h of illumination. Further, using microscale measurements, we identify pre-existing iodide-rich domains as key contributors to both the observed transient photostability and permanent iodine loss in MAPbBrI_2_, and see evidence that extended light soaking results in iodide redistribution that improves optoelectronic homogeneity. Overall, our results emphasize the importance of carrier-induced halide oxidation in creating a dynamic defect landscape in mixed-halide perovskites and provide a framework for interpreting apparent light-driven changes in optoelectronic behavior through the lens of permanent compositional changes.

## Graphical abstract

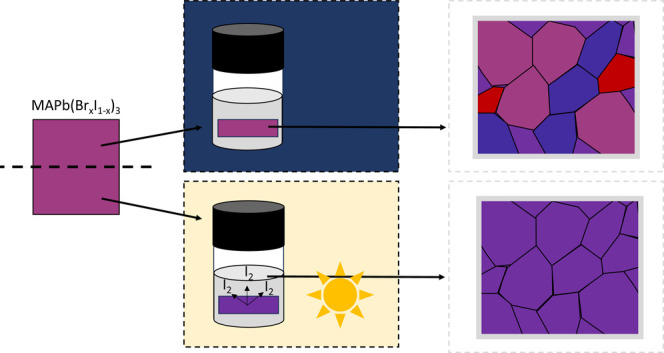

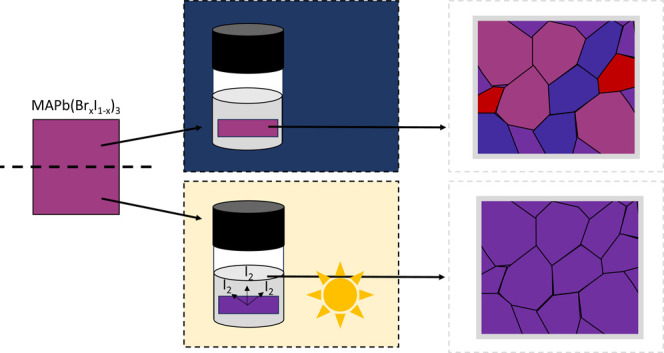

## Introduction

Stability remains a key concern in the development of lead halide perovskites for optoelectronic devices,^1,2^ with substantial research effort focused on improving device architecture, engineering interfaces, and optimizing perovskite chemistries with the aim of achieving long-term performance stability.^3–6^ Perovskites are extremely defect-tolerant,^7^ a key to their success as solution-processed semiconductors, and the presence of ionic defects that can readily move through the lattice at room temperature has been attributed to both reversible changes in behavior (i.e., current–voltage hysteresis and photoinduced halide segregation), and irreversible changes in composition and performance (i.e., electrode corrosion).^8–13^ To add to this dynamic behavior, light-driven photooxidation has been observed to change the number and chemistry of mobile defects in materials, exacerbating instabilities under illumination.^14–17^ Despite these various instabilities having a common origin, the relationships between these instabilities—such as how a dynamic defect landscape changes perovskite structure and composition, and when those changes become permanent—have been under-explored. To develop stable photovoltaics and other optoelectronic devices requires understanding these connections and ultimately controlling these effects.

Light-induced effects on lead halide perovskites are well documented in the literature^18^ and have been observed to be dependent upon perovskite chemistry,^19,20^ illumination conditions,^21,22^ and environment (e.g., in the presence of oxygen or not).^23,24^ In pure halide compositions, such as methylammonium lead iodide and methylammonium lead bromide (methylammonium = MA), light soaking has resulted in both observed photodarkening and photobrightening behavior under illumination.^21,23,25,26^ These differences have been attributed to differences in wavelength and environment, where in one case high-energy photons drive photolysis of PbI_2_ and the subsequent photodegradation of MAPbI_3_, and in the other photon-induced self-healing via ion movement and defect passivation.^21,27^ Beyond photoluminescence, large changes in ionic conductivity have been observed for iodide-based perovskites and attributed to the reversible formation of carrier-induced defects, which can ultimately drive permanent performance changes.^16,17^

For mixed-halide perovskites, their mixed nature drives additional light-induced transient and permanent effects. The well-studied phenomenon of photoinduced halide segregation results in the reversible formation of separate iodide-rich and bromide-rich perovskite phases under illumination.^13,28–30^ These iodide-rich low energy domains limit the quasi-Fermi level splitting within mixed-halide perovskites and ultimately the performance of photovoltaic devices.^31,32^ Additionally, extended light soaking of mixed-halide perovskites has been demonstrated to drive permanent photodecomposition at high fluences,^33^ or the loss of iodine at nearer to 1 sun conditions.^34,35^

Recently, local compositional heterogeneity has emerged as a key to understanding and predicting the photostability of mixed-halide perovskites.^36,37^ However, the literature again presents conflicting narratives on the impacts of light soaking in these materials, where illumination has been observed to drive increased local heterogeneity and subsequent photodegradation in some cases^36,38^ and chemical homogenization and performance improvements in others.^39^

Taken together, these data present a conflicting picture of carrier-induced instabilities in perovskites and point toward a key unresolved question in the development of stable perovskite films. Namely, how do transient light-induced changes in perovskite chemistry, structure, and property relate to permanent changes in composition and performance? Here, we address this question in a model MAPb­(Br_*x*_I_1–*x*_)_3_ system. By combining bulk structural and optical measurements with microscale spectroscopy, we investigate the role of mobile halide defects and local chemistry on reversible and long-term instabilities in these materials.

## Experimental Methods

### Perovskite Sample Preparation

Perovskite thin films on glass substrates were prepared via spin coating in a nitrogen atmosphere. Perovskite solutions were prepared by stoichiometric mixing of 1 M solutions of MAPbI_3_ and MAPbBr_3_ (PbI_2_, TCI; PbBr_2_, TCI; CH_3_NH_3_I, Greatcellsolar materials; CH_3_NH_3_Br, Greatcellsolar materials) in a 3:1 solvent mixture of DMF/DMSO. Solutions were filtered through a 0.2 μm PVDF filter. Before spin coating, substrates were prepared by consecutive sonication in Extran (EMD, EX0996-1), DI water, acetone, and isopropanol. Substrates were then oxygen plasma cleaned immediately before perovskite deposition. Perovskite film deposition and formation were done via the following multistep process: deposition of 50 μL of perovskite solution onto the glass substrate; 20 s of spinning at 6000 rpm (10 s for the MAPbBr_3_ films); immediate immersion into an anisole bath for 30 s; 20 s of spinning at 6000 rpm to dry the film; and 10 min of annealing at 100 °C.

To reduce uncertainty from processing variability, individual perovskite samples were cracked into two pieces and subjected to their respective aging procedures. Samples were either placed into separate scintillation vials, coated with anhydrous toluene (2 mL), and sealed in a nitrogen glovebox, or sealed using UV-cured epoxy (Blufixx Us-10-111-0000) in a nitrogen glovebox. Samples were then placed under a light source (light-sample) or under aluminum foil (dark-sample). For XRD, photothermal deflection spectroscopy (PDS) and absorption measurements samples were exposed to 24 h of ∼450 nm LED illumination. For photoluminescence and hyperspectral measurements, a calibrated solar simulator with an output power of 1 sun was used, and samples were kept at room temperature under 1 sun using active cooling.

### Toluene Absorption

Toluene from aging experiments was collected and transferred to quartz cuvettes. Absorbance data were collected using an Agilent-Cary 100 UV–vis spectrophotometer. A toluene reference was used for background subtraction to isolate the absorbance from the dissolved species.

To qualitatively assess the amount of iodine in solution, background-corrected intensity at 305 and 500 nm was determined. The relative intensity of a sample aged in the light in comparison to a sample aged in the dark for a given experiment (samples from the same original film) was then compared to determine the percent change in absorbance with light soaking.

### Photothermal Deflection Spectroscopy

PDS measurements were conducted using a home-built instrument, as previously described.^40^ Briefly, the pump beam is monochromatic light from a 100 W halogen lamp, selected using an automated monochromator equipped with an order-sorting filter wheel. The pump beam was chopped at 0.5 Hz using a mechanical chopper. This beam was split and separately focused onto both the sample and a reference pyroelectric detector. Samples were loaded air-free within a glovebox into a sealed sample chamber filled with degassed and filtered perfluorohexane (C6F14, 3 M Fluorinert FC-72) as the deflection medium. The probe beam, generated by a HeNe laser, was aligned perpendicular to the pump beam and parallel to the sample surface. Deflection of the probe beam was measured by a position-sensitive Si detector using a lock-in amplifier. Urbach energy was estimated by fitting the observed exponential regime in the PDS signal, and the optical bandgap was estimated by extrapolation of the absorption edge.

### X-ray Diffraction

X-ray diffraction data was collected in the grazing-incidence geometry at beamline 11-3 of the Stanford Synchrotron Radiation Lightsource. Two-dimensional scattering was collected with monochromatic 12.7 keV X-rays and recorded on a Rayonix MX-225 detector measuring 225 × 225 mm^2^. Samples were measured in a chamber under flowing helium. All 2D images were calibrated with a LaB_6_ standard to determine experimental geometry and normalized for incident beam intensity. The pyFAI and pygix Python packages were used for data analysis. Solid angle correction of 2D images was performed in pygix. 1D diffraction patterns were produced by full azimuthal integration using the Python packages pyFAI and pygix. Lattice parameter was determined by fitting diffraction peaks to pseudo-Voigt peak profiles and performing a least-squares regression of the fit peak position. In-plane (IP) versus out-of-plane (OOP) diffraction was assessed by comparing azimuthal integration of the 2D scattering images from a χ-angle of 0° to 30° (OOP) versus 60° to 90° (IP). For plots of intensity versus scattering angle, diffraction across the entire pseudocubic (100) perovskite peak was integrated with appropriate background subtraction. The resultant intensity as a function of chi was further scaled by sin­(χ) to account for scattered material in the grazing-incidence geometry.

### Photoluminescence

Photoluminescence evolution of encapsulated mixed-halide perovskite samples was measured before and after aging. Samples were irradiated with 3 suns (unless otherwise noted) equivalent of 405 nm continuous radiation. Sample emission was continuously monitored by using an HRS-500 spectrograph equipped with a PIXIS:1024BR_eXcelon digital CCD camera. The photoluminescence center of mass wavelength over time was determined by taking the intensity-weighted mean emission wavelength for each spectrum.

### Hyperspectral Imaging

Hyperspectral imaging was done on a photon etc. IMA hyperspectral microscopy. Images were collected with a Zeiss LD Plan-Neofluar 63*x*/0.75 objective with a Hamamatsu ORCA Flash 4.0 V3 CMOS Camera: Hamamatsu ORCA Flash 4.0 V3 a 1024 × 1024 6.5 × 6.5 μm pixel array. Samples were first imaged in reflection and transmission with illumination from a halogen lamp to determine the absorption. Samples were then illuminated with 1 sun equivalent of 405 nm continuous-wave laser epi-illumination. Samples were illuminated for 10 min before photoluminescence spectra were collected. This was done to ensure that perovskite samples had an opportunity to halide segregate before (versus during) photoluminescence data collection. After photoluminescence mapping, samples were again imaged in reflection and transmission with illumination from a halogen lamp to determine final absorption. Spectra were collected and calibrated as previously described to determine the percent absorption and absolute photoluminescence intensity.^37^

For bandgap maps, the local bandgap was determined from a single-onset Tauc fitting method. Data quality was improved by binning adjacent pixels (8 × 8) and fitting the average absorption spectrum to a single bandgap value. For photoluminescence maps, the center of mass emission energy was calculated by taking the photoluminescence intensity-weighted average of emission wavelengths for each spectrum.

Fiducial markers were used to compare the mapped regions before and after light exposure. Images were then correlated, and spectra were compared using the cv2 Python package for image registration.

## Results and Discussion

### Photodriven Iodine Loss in MAPb­(Br_*x*_I_1–*x*_)_3_ to Halide Sink

To evaluate the impacts of light soaking on lead halide perovskites, the effects of aging on solution-processed MAPb­(Br_*x*_I_1–*x*_)_3_ perovskite films were investigated. All samples were made via an anisole-antisolvent deposition process, chosen for its wide-processing window, compatibility with mixed-halide compositions, and demonstrated success in making high-quality perovskite films.^41,42^ To minimize sample-to-sample variation, light-aged and dark-aged samples were prepared from the same original perovskite film (broken into two pieces). Samples were then submerged in anhydrous toluene and aged for 24 h, following established precedent,^17,43^ to monitor changes in the film chemistry as well as identify species expelled into solution. Changes in the thin-film structure and chemistry were evaluated using grazing-incidence wide-angle X-ray scattering (GIWAXS) and PDS. The structural changes observed for the MAPb­(Br_*x*_I_1–*x*_)_3_ compositions are highlighted in Figure 1.

**Figure 1:**
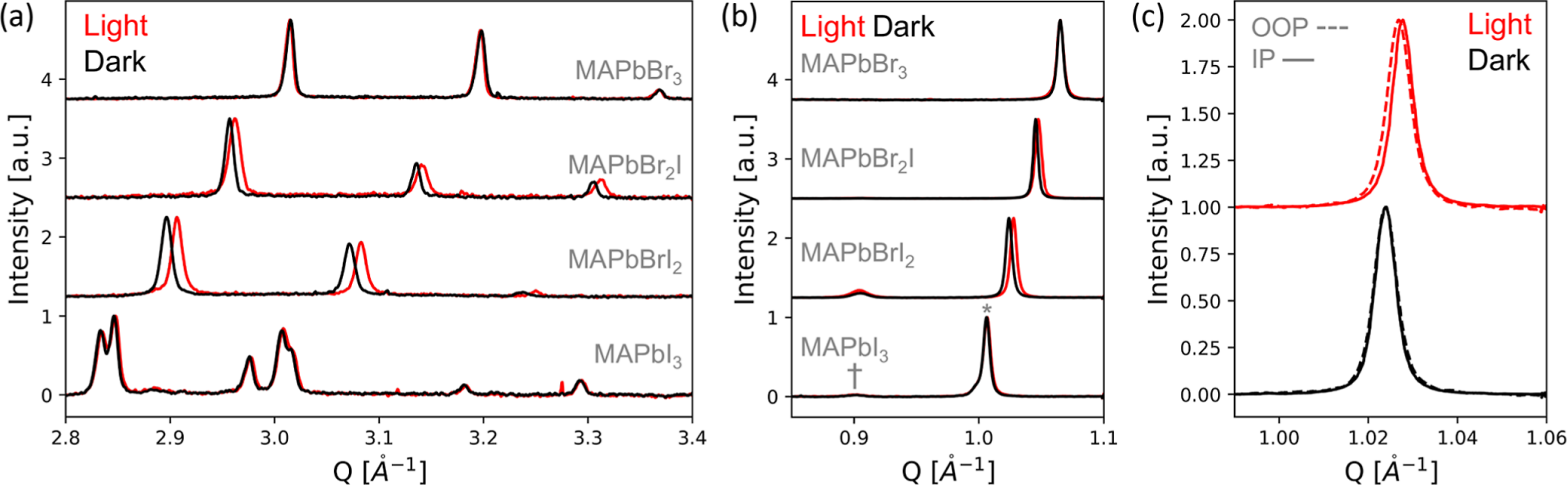
Impact of 24 h ∼ 0.1 sun of 450 nm light exposure on the structure of MAPb­(Br_*x*_I_1–*x*_)_3_ perovskites, showing: (a) normalized diffraction patterns after exposure to light (red) or dark (black) conditions; (b) normalized diffraction from lead iodide (†) and perovskite phases (*); and (c) normalized IP versus OOP diffraction for aged MAPbBrI_2_ thin films.

For all samples, the soaking in toluene itself does not impact the structure (Figure S1). However, 24 h of just ∼0.1 sun illumination induces structural changes in the perovskite samples, specifically the mixed-halide compositions. Figure 1a shows the normalized integrated 1D diffraction pattern for the MAPb­(Br_*x*_I_1–*x*_)_3_ compositions. While the peak shape and position for the pure halide compositions are largely unaffected by illumination, the mixed-halide samples show a noticeable shift in peak position consistent with a contraction of the perovskite lattice. Assuming a cubic lattice for the mixed-halide compositions, the perovskite lattice parameter shrinks by a few hundredths of an Angstrom (from 6.134 Å to 6.114 Å for MAPbBrI_2_ and from 6.010 Å to 5.999 Å for MAPbBr_2_I).

Notable in the GIWAXS data is the overall lack of obvious illumination-driven degradation of a perovskite phase. As highlighted in Figure 1b, we do not observe a consistent increase in the amount of lead iodide in the sample (peak at *q* = 0.903 Å^–1^), as would be expected for degradation via the well documented light-driven decomposition of MAPbI_3_,^44^ suggesting additional PbI_2_ is either amorphous or below our detection limit. Nor do we observe any change in crystallographic orientation as a function of aging condition, though the samples themselves do possess differences in texture (Figure S2). When comparing integrated intensity across samples, despite being two-halves of the same spin-coated sample, we note a reduction in diffracted intensity from the perovskite phase for all iodine-containing samples exposed to light, which would be consistent with some loss of the perovskite crystalline phase with illumination (Figure S3). This decrease in diffracted intensity is the largest for the MAPbBrI_2_ sample, which also showed the largest light-driven lattice contraction.

Beyond the observed shift in the diffraction pattern, the impact of aging on thin-film stress was considered. 2D diffraction images were analyzed to resolve the IP versus OOP diffraction contributions. Figure 1c shows the IP and OOP diffraction for MAPbBrI_2_ films aged under light or dark conditions. All of the light-aged samples show a difference in IP versus OOP diffraction that could be attributed to increased strain in the materials with illumination (Figures S4 and S5). These data support a structural picture of the mixed-halide films where a bulk lattice contraction has occurred and an increase of substrate-induced strain has accompanied that contraction.

All of these structural observations—the changes in lattice parameter, diffracted intensity, and strain—point toward substantial photodriven iodine expulsion from MAPb­(Br_*x*_I_1–*x*_)_3_ perovskites. Carrier-driven halide oxidation that results in halogen loss from the perovskite phase is now well documented in the literature,^17,34,35,45–47^ and in a mixed-halide system, the most easily oxidizable halide (i.e., iodide in an iodide-bromide perovskite) is expected to dominate this reaction.

Recent works by Lee et al. and Xu et al. showing photodriven iodine expulsion from mixed iodide-bromide perovskites under 1 sun and UV-illumination have suggested a photoinduced iodine-loss process in these materials that is not dependent on the formation of PbI_2_, but rather described by the defect reaction shown in eq 1, where *I*_I_ is an occupied iodide site, *V*_I_^•^ is an iodine vacancy, *e*′ is an electron, and *I*_2_ is molecular iodine.^34,35^

1 $$2I_{I}↔2V_{I}^{\cdot }+2e^{\prime }+I_{2}$$

Here, we see strong structural evidence of this phenomenon even at these comparably low light intensities. What is notable in this case is the scale of iodine loss under illumination and the maintained structural quality of a single perovskite phase. Applying Vegard’s law, we estimate an absolute decrease in iodine of 3–5% for our mixed-halide samples (see Supporting Information, Note 1, for details of approximation). This reflects a substantial change to the perovskite chemistry and one, following eq 1, is likely to impact the optoelectronic performance of devices made with such materials as an increased defect and carrier population may be accommodated under illumination (see Supporting Information, Note 2, for further discussion of reaction products).

Our GIWAXS results suggest that mixed-halide perovskites are uniquely susceptible to photodriven iodine loss. To confirm and quantify the impact of this compositional change, the optical properties of both the perovskite film and the aging solution were analyzed. The impact of aging in anhydrous toluene on the film and solution absorption is highlighted in Figure 2.

**Figure 2:**
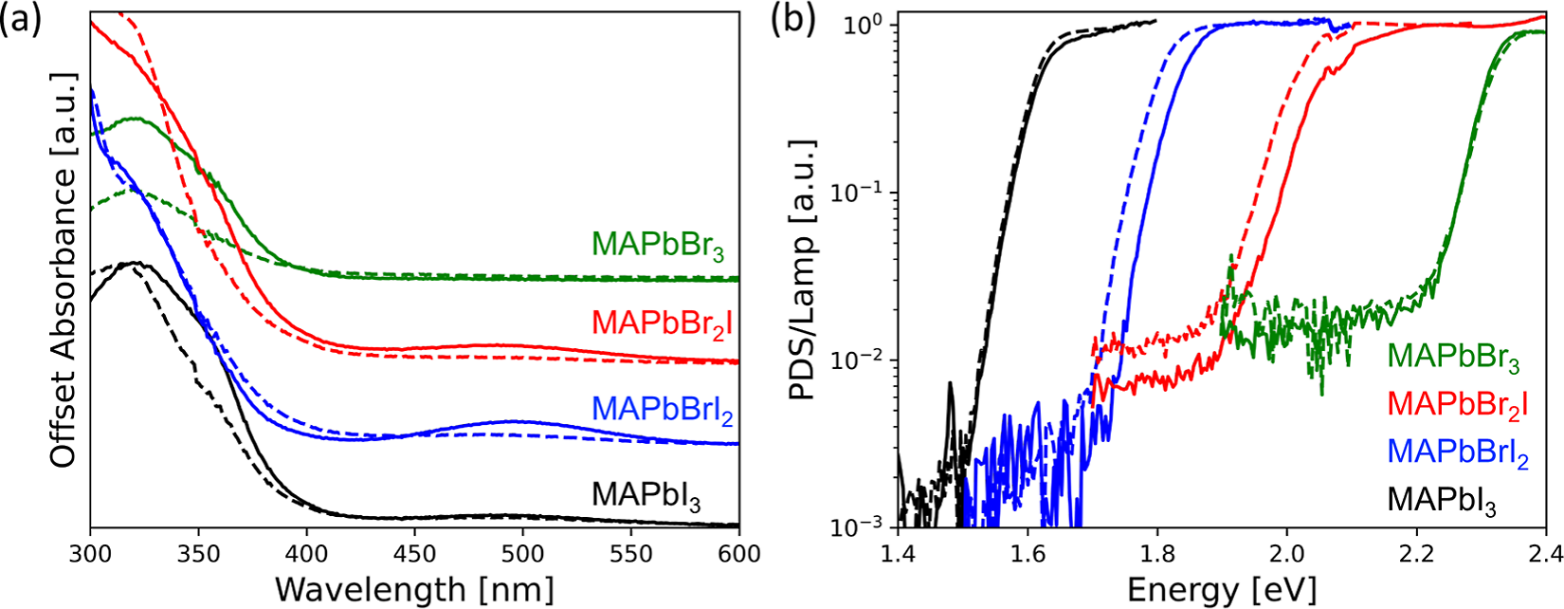
Effects of 24 h of light exposure on absorption of MAPb­(Br_*x*_I_1–*x*_)_3_ perovskites in anhydrous toluene showing: (a) absorbance of toluene liquid for samples aged in the dark (dashed lines) and light (solid lines); (b) PDS results for perovskites after aging in the dark (dashed lines) and light (solid lines).

Analysis of the toluene solution that each film was soaked in shows results consistent with photodriven iodine loss from the perovskite films (Figure 2a). We see an increase in absorbance around 500 nm and in the UV region for the iodine-containing light-soaked samples. These increases in absorbance are consistent with the presence of I_2_ in solution.^17,43,48^ Focusing on the absorption feature around 500 nm, we see notably higher absorbance for the solutions containing the light-soaked mixed-halide samples (∼6 times that of the pure-iodide sample), suggesting that the mixed-halide samples are particularly sensitive to photodriven iodine loss. Using a Beer–Lambert approximation, we estimate an iodine loss from the mixed-halide films into solution on the order of a few percent (see Supporting Information, Note 3, for details of approximation), consistent with our GIWAXS results and suggesting that in the presence of an iodine sink the majority of photodriven iodine lost from the perovskite is expelled into solution instead of being trapped or redistributed in the film. While the magnitude of these absorption changes did vary from sample to sample (suggesting varied amounts of evolved iodine or related species), we see a consistent increase in absorbance for light treated samples across multiple experiments (Figure S6). We also see an increase in UV-absorbance for MAPbBr_3_ solutions, which we attribute to bromine loss.^49^

For the perovskite films themselves, changes in optical bandgap and disorder were assessed via PDS (Figure 2b). The Urbach energy (*E*_U_) was estimated as a measure of electronic disorder for aged perovskite films. By fitting the exponential region of the absorption edge, we observe that all MAPb­(Br_*x*_I_1–*x*_)_3_ samples have Urbach energies in the range of approximately 20–40 meV after being aged in toluene (Figure S7). The Urbach energy is lowest for MAPbI_3_ (19.4 meV) and increases with Br alloying for the mixed halides (a maximum of 35.8 meV for MAPbBr_2_I). These values and trends (increasing *E*_U_ with Br alloying) are similar to previous reports using similar characterization methods,^50,51^ suggesting that the toluene soaking does not substantially degrade or increase the disorder of the perovskite film. Furthermore, in line with our GIWAXS observations, the light soaking does not appear to degrade the optoelectronic quality of the perovskite film: all the light-soaked samples have similar or slightly lower Urbach energies than the samples aged in the dark.

What is affected by light soaking is the absorption onset for the mixed-halide films. For the mixed-halide perovskites, there is a clear blue shift in the PDS spectra with light soaking (Figure 2b), indicating an increase in the optical band gap. We estimate a bandgap increase of approximately 30 meV for the MAPbBrI_2_ and MAPbBr_2_I samples with light soaking. This increase corresponds to a decrease of iodide fraction of approximately 3–4 percentage points (see Supporting Information, Note 1, for details of approximation) and is again in good agreement with our GIWAXS results.

Taken together, the GIWAXS and absorption results suggest that mixed-halide perovskites, well-known for instabilities related to carrier-driven halide segregation,^13,42^ are also uniquely susceptible to carrier-driven halide oxidation and expulsion. While iodine expulsion in mixed-halide perovskites has been previously observed,^34,35^ here we note the unique susceptibility of mixed halides to selective iodine loss and additionally observe that this loss leaves behind a bromide-enriched perovskite with structural and electronic disorder comparable to those of the original film. Despite a significant change in composition, we did not see significant evidence of increased compositional or structural heterogeneity.

While these experiments were conducted under low-illumination conditions and in the presence of an iodine sink, the carrier-driven halide oxidation and expulsion observed are likely relevant in mixed-halide perovskites used in device configurations. While the lack of an iodine sink will change the iodine loss pathways, commonly used organic contact layers are not perfect halide-permeation barriers.^52^ As such, we anticipate the substantial carrier-driven iodine loss seen here to manifest as iodide redistribution or continued iodine loss under more device-relevant illumination conditions, i.e., even in the presence of an encapsulation layer. To probe these effects and assess the impact of light soaking on mixed-halide perovskites in the absence of an iodine sink, additional measurements on encapsulated samples aged under 1 sun illumination were evaluated.

### Photodriven Changes to Optoelectronic Performance and Heterogeneity in Encapsulated Films

Films for PL measurements were encapsulated prior to aging under 1 sun to maintain a continuous inert environment for the duration of the test and probe the impacts of light-soaking in the absence of an iodine sink. We expect these changes to affect the dynamics of halide evolution from the samples—as the illumination intensity is increased and there is no longer a toluene sink to absorb iodine species—and the iodine evolution pathways, where instead iodine (if evolved) may be absorbed into the epoxy or may be redistributed through the perovskite films itself. The results of aging under 1 sun illumination on the PL of encapsulated MAPbBrI_2_ films are presented in Figure 3.

**Figure 3:**
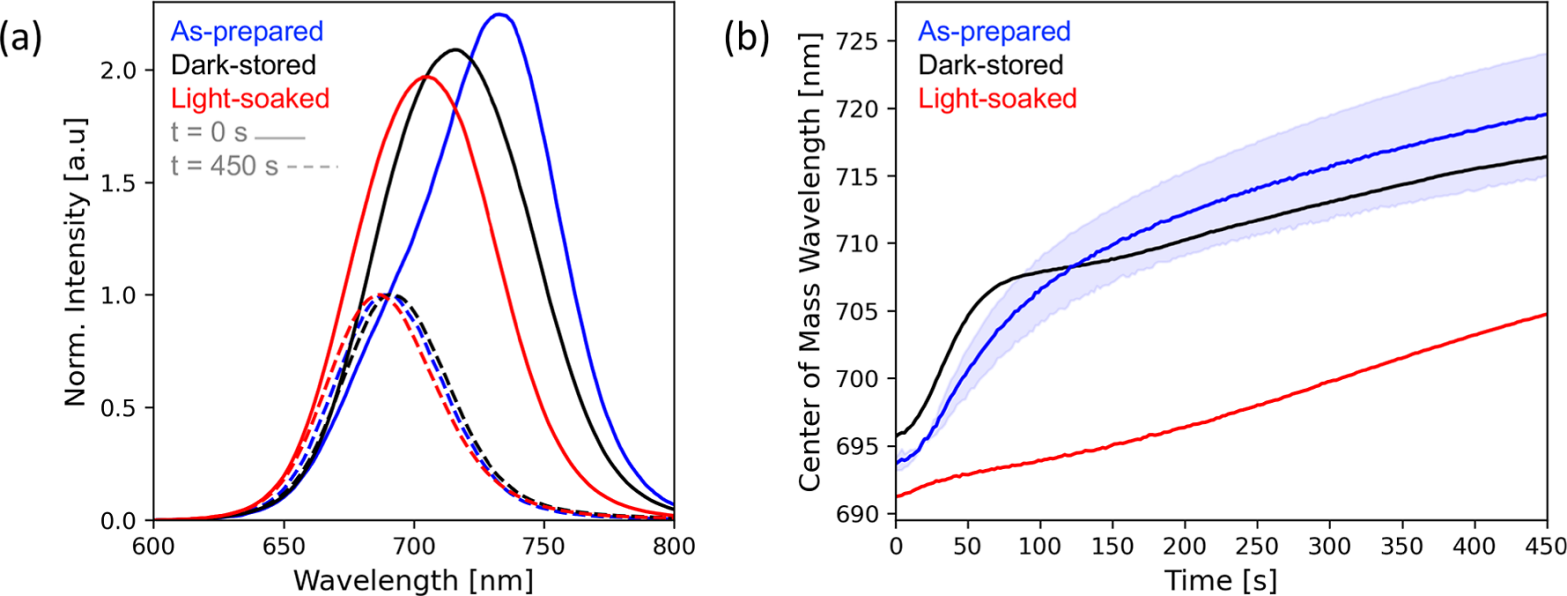
Effect of aging on encapsulated MAPbBrI_2_ films on photoluminescence. (a) Impact on photoluminescence spectra comparing as-prepared films (blue), dark-stored films (black), and films light-soaked with 1 sun of AM1.5 (red) for 48 h highlighting initial spectra (dashed lines) and spectra after 450 s (7.5 min) of ∼3 suns equivalent of 405 nm CW laser illumination (solid lines). (b) Evolution of the intensity-weighted mean photoluminescence wavelength (center of mass wavelength) with ∼3 suns equivalent of 405 nm CW laser illumination for as-prepared (blue) dark-stored (black) and light-soaked with 1 sun of AM1.5 (red) for 48 h samples. Shaded blue region shows range of response for as-prepared samples.

While absorption provides information about the bulk bandgap of these films, carrier funneling into low bandgap phases allows us to see trace amounts of these phases via photoluminescence. Looking at the PL spectra of the encapsulated MAPbBrI_2_ samples (Figure 3a), the light-soaked samples have an initial PL peak that is blue-shifted with respect to the as-prepared film and dark-stored film (∼10 meV). This observation is consistent with an observed shift in the absorption onset (Figure S8). These results again support photodriven iodine loss from the MAPbBrI_2_ perovskite phase, despite encapsulation. We note that we do not observe this trend for the encapsulated MAPbBr_2_I samples, where there is variation in the initial PL peak across all samples (Figure S9). We attribute some of these differences in PL spectra to the sensitivity of PL to low bandgap domains and a potentially more complicated iodide redistribution process in encapsulated films.

In addition to the initial PL spectra, we also monitor its time evolution. Both photodriven iodine expulsion and halide segregation, where excited state carriers drive the formation of low-bandgap iodide-rich phases in mixed-halide perovskites, have been linked to the oxidation of iodide in the perovskite lattice.^15,17,34,35^ As such, we anticipate extended light soaking and the resulting permanent iodine expulsion or rearrangement could affect the reversible halide segregation process. In analyzing the PL of our encapsulated samples, we see evidence of such a relationship. We observe a substantial difference in the center of mass of the PL spectra for various aging conditions. After extended light soaking, MAPbBrI_2_ samples show a higher energy and substantially slower change in PL center-of-mass when compared to the PL transient of the as-prepared samples or that of samples stored in the dark (Figures 3b and S10). This is opposite to the generally observed trend, where an increase in bandgap from increased bromide fraction results in a faster halide-segregation process.^30^ In comparing the light-soaked film to the as-prepared sample, the PL center-of-mass red shifts to 1.75 eV versus 1.72 eV in 7.5 min, suggesting that the 1 sun illumination has improved stability to halide segregation under subsequent laser illumination. We observe a similar dynamic in the MAPbBr_2_I samples (Figure S9), where the PL transients of as-prepared and dark-stored samples are qualitatively similar to each other, showing a rapid initial red shift followed by a blue shift within the first ∼10 s of illumination, but distinct from the PL transient of the light-soaked sample.

To connect the observed changes in PL to the process of halide oxidation and redistribution, we look to optoelectronic property mapping on the micrometer scale. Prior reports of PL mapping of MAPbI_3_ films have revealed that extended light soaking drives the redistribution of iodide from iodide-rich to iodide-poor areas within the perovskite, improving compositional and optoelectronic homogeneity of the perovskite film.^27,53^ To see if a similar effect is occurring in these mixed-halide samples, driving both the observed compositional changes and stability improvements, we collected absorption and photoluminescence maps both before and after aging. Absorption was measured to qualitatively assess the local composition of the film, while PL was collected to assess optoelectronic performance and stability. The data collection procedure was designed with the aim of distinguishing between what are considered largely short-term effects of light soaking (e.g., photobrightening and halide segregation) and permanent changes in structure and composition (e.g., iodine expulsion). The impact of both short-term light soaking (1 sun equivalent of 405 nm CW laser excitation for tens of minutes) and extended light soaking (100 mW/cm^2^ of AM1.5 for 48 h) on local composition is highlighted in Figure 4.

**Figure 4:**
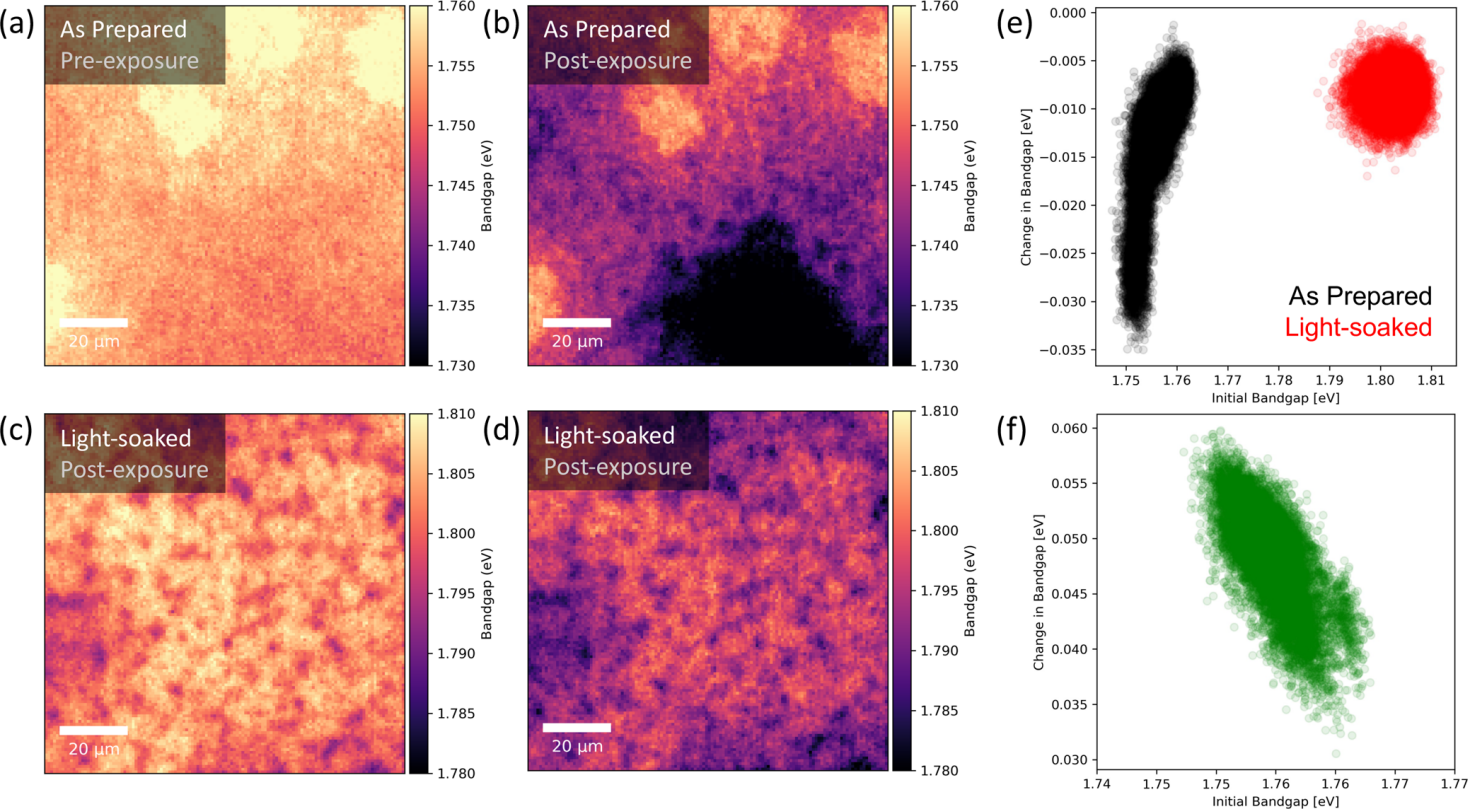
Impact of light soaking on optical bandgap derived from Tauc method of local absorption spectra for an encapsulated MAPbBrI_2_ film. Maps of optical bandgap for as-prepared sample before (a) and after (b) ∼60 min of ∼1 sun equivalent of 405 nm CW laser light exposure; and sample light-soaked with 1 sun of AM1.5 for 48 h before (c) and after (d) similar 405 nm light exposure. (e) Correlation of initial bandgap (data in (a,c)) and bandgap change (in comparison to (b,d), respectively) during ∼60 min of ∼1 sun equivalent of 405 nm CW laser light exposure for as-prepared (black) and light-soaked (red) samples. (f) Correlation of initial bandgap (data in (a)) and bandgap change (in comparison to (b)) after 1 sun of AM 1.5 for 48 h.

Our mapping reveals a MAPbBrI_2_ perovskite film with heterogeneous and dynamic optoelectronic properties under illumination, and by mapping the behavior over time, we can identify pre-existing iodide-rich domains as a substantial source of instability. We first look at as-prepared MAPbBrI_2_ films and changes in optoelectronic property with exposure to ∼60 min of 1 sun equivalent of 405 nm CW laser illumination. We see evidence of compositional heterogeneity even before any light soaking has occurred (Figure 4a). Assuming that bandgap fluctuations as inferred from local absorption measurements come from variations in local halide fraction, this represents a slight initial variation in halide distribution in our perovskite film. This heterogeneity is further exacerbated with exposure to ∼60 min of 1 sun equivalent of 405 nm CW laser illumination (Figure 4b), where illumination is observed to drive the formation of perovskite phases with even lower bandgaps, as expected from halide segregation.^28^ While the bandgap decreases across the whole film, suggesting some amount of halide segregation throughout the film with the iodide-rich domains dominating our absorption observations, we observe a strong correlation between initial bandgap (Figure 4a) and magnitude of bandgap change. We see that the areas that have the lowest initial bandgaps (a.k.a. more iodide-rich) experience the largest decrease in bandgap with the 405 nm light exposure (Figure 4e).

After this initial mapping, which has already changed the apparent compositional heterogeneity of the film, the samples are light-soaked under 1 sun for 48 h before being reanalyzed. Here we observe that this extended illumination substantially changes the morphology, the band gap, and the stability of the film, and we can again link these changes to the original iodide-rich domains. In looking at the absorption of the film, we find that the morphology of the film has changed, while the overall bandgap of the imaged area has increased (Figure 4c). In comparing this aged map (Figure 4c) to the original behavior (Figure 4a), we again observe a correlation between initial bandgap and magnitude of bandgap change (Figure 4f). We observe a negative correlation, where areas with smaller initial bandgaps experience larger changes in bandgap over prolonged illumination.

For the light-soaked samples, we again expose them to ∼60 min of 1 sun equivalent of 405 nm CW laser illumination and we again see an overall reduction in bandgap (Figure 4d). However, in comparison to the as-prepared samples, the impact of the subsequent light exposure is suppressed and largely independent of the initial bandgap (Figure 4e). Taken together, these imaging results suggest that heterogeneity, namely, the preexistence of iodide-rich domains, is a source of both short-term (e.g., photoinduced halide segregation) and long-term (e.g., permanent bandgap changes) instabilities.

Finally, our mapping results also suggest that extended light soaking substantially changes the low-band gap iodide-rich regions that dominate subsequent light-induced optical response. Not only do we observe more stable bandgaps (as monitored by absorption) after 48 h of 1 sun illumination, but we also see substantially more homogeneous PL emission after aging. Looking at PL maps before (Figure 5a) and after (Figure 5b), we see a substantial homogenization of emission energy around 1.69 eV. By comparing these maps to maps of PL intensity (Figure S11), we can confirm that this improvement in homogeneity, which has been linked to improved device stability,^36,38^ does not come at the cost of PL emission intensity.

**Figure 5:**
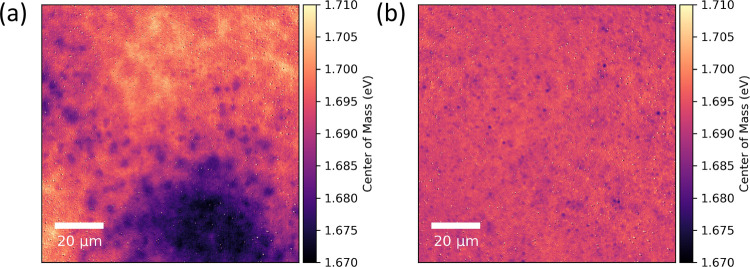
Impact of light soaking on photoluminescence spectra. Maps of photoluminescence spectra center of mass for a MAPbBrI_2_ film as-prepared (a) and after (b) 48 h 100 mW/cm^2^ of AM 1.5 illumination.

Overall, using a range of bulk and microscale techniques, we see abundant evidence that iodide distribution plays an important and dynamic role in the performance of iodide-containing perovskites. For our mixed-halide perovskite, we have demonstrated that light can drive the selective expulsion and redistribution of iodine from the perovskite lattice, resulting in substantial structural and compositional changes. We observe this in the presence of an iodine sink at low illumination intensities and still in an encapsulated film where iodine loss is suppressed at 1 sun. Further, from our mapping results, we can point to the most iodide-rich and least stable perovskite domains within the films as the dominant source of that expelled iodine. We can explain this sensitivity in mixed-halide perovskites by considering the impact of compositional heterogeneity in these materials. Under illumination, mixed-halide perovskites with a variation in halide composition will funnel holes to the lowest band gap, most iodide-rich, domains. This accumulation of holes in the proximity of iodide is expected to accelerate halide oxidation and increase the transport of iodine both out of and within the film.^15,30^ From our work, we observe that the result of this apparent carrier accumulation is iodine redistribution and iodine loss, which can result in improved photostability and PL homogeneity.

Taken together, these results suggest pathways for both developing processing methods to favor stability and evaluating the stability of the new materials. In principle, these results suggest that the fundamental susceptibility of a mixed-halide perovskite to halide oxidation is an important predictor of the compositional and operational stability. As such, 24 h light-aging tests as shown here (i.e., aging in anhydrous toluene) could be a rapid way to screen new materials. Additionally, for materials that experience selective halide oxidation, this work suggests that extended light-soaking and allowing halogens to redistribute may be a pathway toward improved photostability and optoelectronic property homogenization. Recent work has already shown this a promising strategy for improving stability^39^ and may be a pathway for expanding the range of mixed-halide perovskite compositions being developed for optoelectronic devices. Further work should continue to explore the impacts of selective iodine loss and redistribution on device performance, where interactions with contact materials, a dynamic defect population, and a changing carrier population are all likely to impact performance and stability.

## Conclusions

Light-induced instabilities are well documented in MAPbX_3_ perovskites, yet the role of chemistry and time scale on stability have been underexplored. Here, we present direct evidence that the continued illumination of MAPbX_3_ perovskites produces both transient and permanent changes in perovskite structure and optoelectronic performance. By looking at perovskite thin films, we document the photodriven expulsion of halogens from the material into an iodine sink and see evidence of the selective expulsion of iodine without increasing the apparent compositional or electronic disorder of the film. We document this phenomenon with GIWAXS and PDS, measuring 3–5% absolute loss in iodine in mixed-halide perovskites. To better understand the origins and impacts of this iodine redistribution under more device-relevant conditions, we employ photoluminescence and hyperspectral mapping techniques on encapsulated films. Overall, these results reveal pre-existing iodide-rich domains as a dominant source of optoelectronic instability in our mixed-halide perovskites and suggest that controlling these either by process engineering or postprocessing light soaking may be a pathway to improved stability. While our work here focuses on MAPbX_3_ perovskites, it points to the overall importance of understanding the dynamic defect landscape in perovskites, as carrier-driven changes to defect density and distribution are likely to drive variations in composition and performance over time.

## Supplementary Material



## Notes

Supplemental XRD results and analysis; average changes in toluene absorption; PDS spectra with fits to determine Urbach energy; PL and absorption changes under illumination; photoluminescence mapping; and method of estimating iodine loss from XRD, PDS, and absorbance measurements (PDF)

## #

Chemistry Department, Princeton University, Princeton, NJ 08544, United States

## ¶

Materials Science and Engineering Department, Northwestern University, Evanston, IL 60208, United States.

## References

[1] Zhu H., Teale S., Lintangpradipto M. N., Mahesh S., Chen B., McGehee M. D., Sargent E. H., Bakr O. M. (2023). Long-Term Operating
Stability in Perovskite Photovoltaics. Nat.
Rev. Mater..

[2] Ahn N., Choi M. (2024). Towards Long-Term Stable
Perovskite Solar Cells: Degradation Mechanisms
and Stabilization Techniques. Adv. Sci..

[3] Saliba M., Matsui T., Seo J. Y., Domanski K., Correa-Baena J. P., Nazeeruddin M. K., Zakeeruddin S. M., Tress W., Abate A., Hagfeldt A., Grätzel M. (2016). Cesium-Containing
Triple Cation Perovskite
Solar Cells: Improved Stability, Reproducibility and High Efficiency. Energy Environ. Sci..

[4] Cheacharoen R., Boyd C. C., Burkhard G. F., Leijtens T., Raiford J. A., Bush K. A., Bent S. F., McGehee M. D. (2018). Encapsulating Perovskite
Solar Cells to Withstand Damp Heat and Thermal Cycling. Sustainable Energy Fuels.

[5] Grancini G., Roldán-Carmona C., Zimmermann I., Mosconi E., Lee X., Martineau D., Narbey S., Oswald F., De Angelis F., Graetzel M., Nazeeruddin M. K. (2017). One-Year Stable Perovskite Solar
Cells by 2D/3D Interface Engineering. Nat. Commun..

[6] Beal R. E., Slotcavage D. J., Leijtens T., Bowring A. R., Belisle R. A., Nguyen W. H., Burkhard G. F., Hoke E. T., Mcgehee M. D. (2016). Cesium
Lead Halide Perovskites with Improved Stability for Tandem Solar Cells. J. Phys. Chem. Lett..

[7] Steirer K. X., Schulz P., Teeter G., Stevanovic V., Yang M., Zhu K., Berry J. J. (2016). Defect
Tolerance
in Methylammonium Lead Triiodide Perovskite. ACS Energy Lett..

[8] Unger E. L., Hoke E. T., Bailie C. D., Nguyen W. H., Bowring A. R., Heumüller T., Christoforo M. G., McGehee M. D. (2014). Hysteresis and Transient
Behavior in Current–Voltage Measurements of Hybrid-Perovskite
Absorber Solar Cells. Energy Environ. Sci..

[9] Bi E., Song Z., Li C., Wu Z., Yan Y. (2021). Mitigating
Ion Migration in Perovskite Solar Cells. Trends
Chem..

[10] Snaith H. J., Abate A., Ball J. M., Eperon G. E., Leijtens T., Noel N. K., Stranks S. D., Wang J. T.-W., Wojciechowski K., Zhang W. (2014). Anomalous Hysteresis
in Perovskite Solar Cells. J. Phys. Chem. Lett..

[11] Belisle R. A., Nguyen W. H., Bowring A. R., Calado P., Li X., Irvine S. J. C., McGehee M. D., Barnes P. R. F., O’Regan B. C. (2017). Interpretation
of Inverted Photocurrent Transients in Organic Lead Halide Perovskite
Solar Cells: Proof of the Field Screening by Mobile Ions and Determination
of the Space Charge Layer Widths. Energy Environ.
Sci..

[12] Lin C.-H., Hu L., Guan X., Kim J., Huang C.-Y., Huang J.-K., Singh S., Wu T. (2022). Electrode
Engineering in Halide Perovskite
Electronics: Plenty of Room at the Interfaces. Adv. Mater..

[13] Hoke E. T., Slotcavage D. J., Dohner E. R., Bowring A. R., Karunadasa H. I., McGehee M. D. (2015). Reversible Photo-Induced Trap Formation in Mixed-Halide
Hybrid Perovskites for Photovoltaics. Chem.
Sci..

[14] Bertoluzzi L., Patel J. B., Bush K. A., Boyd C. C., Kerner R. A., O’Regan B. C., McGehee M. D. (2021). Incorporating Electrochemical Halide
Oxidation into Drift-Diffusion Models to Explain Performance Losses
in Perovskite Solar Cells under Prolonged Reverse Bias. Adv. Energy Mater..

[15] Kerner R. A., Xu Z., Larson B. W., Rand B. P. (2021). The Role of Halide Oxidation in Perovskite
Halide Phase Separation. Joule.

[16] Kim G. Y., Senocrate A., Wang Y., Moia D., Maier J. (2021). Photo-Effect
on Ion Transport in Mixed Cation and Halide Perovskites and Implications
for Photo-Demixing. Angew. Chem., Int. Ed..

[17] Kim G. Y., Senocrate A., Yang T.-Y., Gregori G., Grätzel M., Maier J. (2018). Large Tunable Photoeffect on Ion Conduction in Halide Perovskites
and Implications for Photodecomposition. Nat.
Mater..

[18] Wang Z., Zhang Z., Xie L., Wang S., Yang C., Fang C., Hao F. (2022). Recent Advances
and Perspectives
of Photostability for Halide Perovskite Solar Cells. Adv. Opt. Mater..

[19] Martani S., Zhou Y., Poli I., Aktas E., Meggiolaro D., Jiménez-López J., Wong E. L., Gregori L., Prato M., Di Girolamo D., Abate A., De Angelis F., Petrozza A. (2023). Defect Engineering to Achieve Photostable Wide Bandgap
Metal Halide Perovskites. ACS Energy Lett..

[20] Knight A. J., Borchert J., Oliver R. D. J., Patel J. B., Radaelli P. G., Snaith H. J., Johnston M. B., Herz L. M. (2021). Halide Segregation
in Mixed-Halide Perovskites: Influence of A-Site Cations. ACS Energy Lett..

[21] Quitsch W.-A., Dequilettes D. W., Pfingsten O., Schmitz A., Ognjanovic S., Jariwala S., Koch S., Winterer M., Ginger D. S., Bacher G. (2018). The Role of Excitation Energy in Photobrightening and
Photodegradation of Halide Perovskite Thin Films. J. Phys. Chem. Lett..

[22] Okrepka H., Ding Y., Ghonge S., Ruth A., Kuno M. (2024). Excitation
Intensity-Dependent Terminal Halide Photosegregation Stoichiometries
in Formamidinium/Cesium Lead Iodide/Bromide [FACsPb­(I1-XBrx)­3] Thin
Films. J. Phys. Chem. Lett..

[23] Brenes R., Eames C., Bulović V., Islam M. S., Stranks S. D. (2018). The Impact
of Atmosphere on the Local Luminescence Properties of Metal Halide
Perovskite Grains. Adv. Mater..

[24] Bryant D., Aristidou N., Pont S., Sanchez-Molina I., Chotchunangatchaval T., Wheeler S., Durrant J. R., Haque S. A. (2016). Light and
Oxygen Induced Degradation Limits the Operational Stability of Methylammonium
Lead Triiodide Perovskite Solar Cells. Energy
Environ. Sci..

[25] Motti S. G., Gandini M., Barker A. J., Ball J. M., Srimath
Kandada A. R., Petrozza A. (2016). Photoinduced Emissive Trap States
in Lead Halide Perovskite Semiconductors. ACS
Energy Lett..

[26] Halder A., Pathoor N., Chowdhury A., Sarkar S. K. (2018). Photoluminescence
Flickering of Micron-Sized Crystals of Methylammonium Lead Bromide:
Effect of Ambience and Light Exposure. J. Phys.
Chem. C.

[27] deQuilettes D. W., Zhang W., Burlakov V. M., Graham D. J., Leijtens T., Osherov A., Bulović V., Snaith H. J., Ginger D. S., Stranks S. D. (2016). Photo-Induced Halide Redistribution in Organic–Inorganic
Perovskite Films. Nat. Commun..

[28] Brennan M. C., Ruth A., Kamat P. V., Kuno M. (2020). Photoinduced Anion
Segregation in Mixed Halide Perovskites. Trends
Chem..

[29] Halford G. C., Deng Q., Gomez A., Green T., Mankoff J. M., Belisle R. A. (2022). Structural Dynamics
of Metal Halide Perovskites during
Photoinduced Halide Segregation. ACS Appl. Mater.
Interfaces.

[30] Suchan K., Just J., Beblo P., Rehermann C., Merdasa A., Mainz R., Scheblykin I. G., Unger E. (2023). Multi-Stage Phase-Segregation of Mixed Halide Perovskites under Illumination:
A Quantitative Comparison of Experimental Observations and Thermodynamic
Models. Adv. Funct. Mater..

[31] Knight A. J., Wright A. D., Patel J. B., McMeekin D. P., Snaith H. J., Johnston M. B., Herz L. M. (2019). Electronic Traps and Phase Segregation
in Lead Mixed-Halide Perovskite. ACS Energy
Lett..

[32] Ruth A., Kuno M. (2023). Modeling the Photoelectrochemical
Evolution of Lead-Based, Mixed-Halide
Perovskites Due to Photosegregation. ACS Nano.

[33] Ruan S., Surmiak M. A., Ruan Y., McMeekin D. P., Ebendorff-Heidepriem H., Cheng Y. B., Lu J., McNeill C. R. (2019). Light Induced Degradation
in Mixed-Halide Perovskites. J. Mater. Chem.
C.

[34] Lee M., Vigil J. A., Jiang Z., Karunadasa H. I. (2025). Evidence
for I2 Loss from the Perovskite-Gas Interface upon Light-Induced Halide
Segregation. Chem. Sci..

[35] Xu Z., Zhong X., Hu T., Hu J., Kahn A., Rand B. P. (2024). Correlating Halide Segregation with Photolysis in Mixed-Halide
Perovskites via In Situ Opto-Gravimetric Analysis. J. Am. Chem. Soc..

[36] Mundt L. E., Zhang F., Palmstrom A. F., Xu J., Tirawat R., Kelly L. L., Stone K. H., Zhu K., Berry J. J., Toney M. F., Schelhas L. T. (2022). Mixing Matters: Nanoscale Heterogeneity
and Stability in Metal Halide Perovskite Solar Cells. ACS Energy Lett..

[37] Frohna K., Anaya M., Macpherson S., Sung J., Doherty T. A. S., Chiang Y. H., Winchester A. J., Orr K. W. P., Parker J. E., Quinn P. D., Dani K. M., Rao A., Stranks S. D. (2022). Nanoscale
Chemical Heterogeneity Dominates the Optoelectronic Response of Alloyed
Perovskite Solar Cells. Nat. Nanotechnol..

[38] Datta K., van Laar S. C. W., Taddei M., Hidalgo J., Kodalle T., Aalbers G. J. W., Lai B., Li R., Tamura N., Frencken J. T. W., Quiroz Monnens S. V., Westbrook R. J. E., Graham D. J., Sutter-Fella C. M., Correa-Baena J.-P., Ginger D. S., Wienk M. M., Janssen R. A. J. (2025). Local
Halide
Heterogeneity Drives Surface Wrinkling in Mixed-Halide Wide-Bandgap
Perovskites. Nat. Commun..

[39] Du L., Cao F., Meng R., Zhang Y., Zhang J., Gao Z., Chen C., Li C., Zhao D., Ye J., Li Z., Xiao C. (2025). Photo-Homogenization Assisted Segregation Easing Technique
(PHASET) for Highly Efficient and Stable Wide-Bandgap Perovskite Solar
Cells. Nat. Commun..

[40] Vandewal K., Albrecht S., Hoke E. T., Graham K. R., Widmer J., Douglas J. D., Schubert M., Mateker W. R., Bloking J. T., Burkhard G. F., Sellinger A., Fréchet J. M. J., Amassian A., Riede M. K., McGehee M. D., Neher D., Salleo A. (2014). Efficient Charge Generation by Relaxed
Charge-Transfer
States at Organic Interfaces. Nat. Mater..

[41] Zhao P., Kim B. J., Ren X., Lee D. G., Bang G. J., Jeon J. B., Kim W. B., Jung H. S. (2018). Antisolvent with
an Ultrawide Processing Window for the One-Step Fabrication of Efficient
and Large-Area Perovskite Solar Cells. Adv.
Mater..

[42] Halford G. C., Deng Q., Gomez A., Green T., Mankoff J. M., Belisle R. A. (2022). Structural Dynamics of Metal Halide Perovskites during
Photoinduced Halide Segregation. ACS Appl. Mater.
Interfaces.

[43] Zhou Y., van Laar S. C. W., Meggiolaro D., Gregori L., Martani S., Heng J.-Y., Datta K., Jiménez-López J., Wang F., Wong E. L., Poli I., Treglia A., Cortecchia D., Prato M., Kobera L., Gao F., Zhao N., Janssen R. A. J., De Angelis F., Petrozza A. (2024). How Photogenerated I2 Induces I-Rich Phase Formation
in Lead Mixed Halide Perovskites. Adv. Mater..

[44] Wang S., Jiang Y., Juarez-Perez E. J., Ono L. K., Qi Y. (2017). Accelerated
Degradation of Methylammonium Lead Iodide Perovskites Induced by Exposure
to Iodine Vapour. Nat. Energy.

[45] Akbulatov A. F., Ustinova M. I., Shilov G. V., Dremova N. N., Zhidkov I. S., Kurmaev E. Z., Frolova L. A., Shestakov A. F., Aldoshin S. M., Troshin P. A. (2021). Temperature Dynamics
of MAPbI3and
PbI2Photolysis: Revealing the Interplay between Light and Heat, Two
Enemies of Perovskite Photovoltaics. J. Phys.
Chem. Lett..

[46] Xu Z., Kerner R. A., Harvey S. P., Zhu K., Berry J. J., Rand B. P. (2023). Halogen
Redox Shuttle Explains Voltage-Induced Halide
Redistribution in Mixed-Halide Perovskite Devices. ACS Energy Lett..

[47] Szabo G., Kuno M., Kamat P. V. (2025). Iodine’s
Wild Ride Leading
to Photoinstability in Halide Perovskite Cells. ACS Energy Lett..

[48] Samu G. F., Balog Á., De Angelis F., Meggiolaro D., Kamat P. V., Janáky C. (2019). Electrochemical
Hole Injection Selectively
Expels Iodide from Mixed Halide Perovskite Films. J. Am. Chem. Soc..

[49] Keefer R. M., Andrews L. J. (1950). The Interaction of Bromine with Benzene and Certain
of Its Derivatives. J. Am. Chem. Soc..

[50] Witt C., Schötz K., Köhler A., Panzer F. (2024). Understanding Method-Dependent
Differences in Urbach Energies in Halide Perovskites. J. Phys. Chem. C.

[51] Sadhanala A., Deschler F., Thomas T. H., Dutton S. E., Goedel K. C., Hanusch F. C., Lai M. L., Steiner U., Bein T., Docampo P., Cahen D., Friend R. H. (2014). Preparation
of Single-Phase
Films of CH_3_NH_3_Pb­(I_1‑X_Br_x_)_3_ with Sharp Optical Band Edges. J. Phys. Chem. Lett..

[52] Kerner R. A., Heo S., Roh K., MacMillan K., Larson B. W., Rand B. P. (2021). Organic
Hole Transport Material Ionization Potential Dictates Diffusion Kinetics
of Iodine Species in Halide Perovskite Devices. ACS Energy Lett..

[53] Kim T., Park S., Iyer V., Shaheen B., Choudhry U., Jiang Q., Eichman G., Gnabasik R., Kelley K., Lawrie B., Zhu K., Liao B. (2023). Mapping the Pathways
of Photo-Induced Ion Migration in Organic-Inorganic Hybrid Halide
Perovskites. Nat. Commun..

