# Supplementary material for: Light-Driven Iodine Loss and Photoluminescence Homogenization in Mixed-Halide Perovskite Semiconductors
Source: J Phys Chem C Nanomater Interfaces. 2026 Jul 8;130(29):10357–66. doi: 10.1021/acs.jpcc.6c02043 (PMC13403311; doi:10.1021/acs.jpcc.6c02043)
Supplement: Supplementary file 1 [file jp6c02043_si_001.pdf]

Light-driven Iodine Loss and Photoluminescence Homogenization in Mixed-Halide Perovskite Semiconductors

Maya J. Lebowitz[a], Annie Gomez[c], Benjamin L. Cotts[d,e], Samuel D. Stranks[f], Alberto Salleo[d], Rebecca A. Belisle[c]*

[a]Chemistry Department, Wellesley College, Wellesley, MA 02481, United States
[c]Department of Physics and Astronomy, Wellesley College, Wellesley, MA 02481, United States
[d]Department of Materials Science and Engineering, Stanford University, Stanford, CA 94305, United States
[e]Department of Chemistry and Biochemistry, Middlebury College, Middlebury, VT 05753, United States
[f]Department of Chemical Engineering and Biotechnology, University of Cambridge, Cambridge CB3 0AS, U.K.

*Email: rbelisle@wellesley.edu

Supporting Information

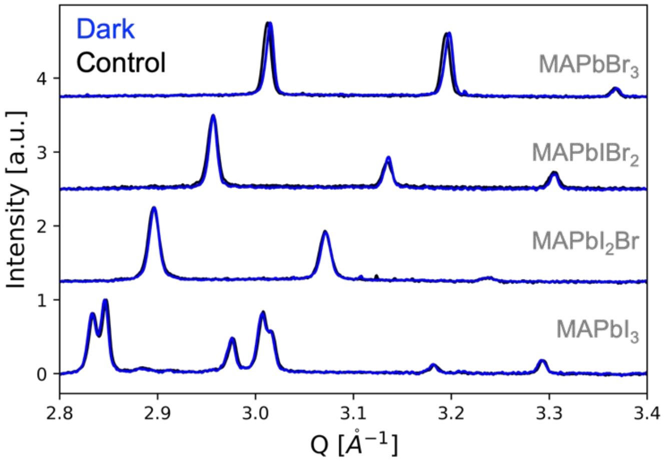

Figure S1: Integrated diffraction patterns for $MAPbX_3$ compositions after 24 hours in toluene in the dark followed by storage in an inert environment (blue), versus no exposure to toluene and continued storage in an inert environment storage (black).

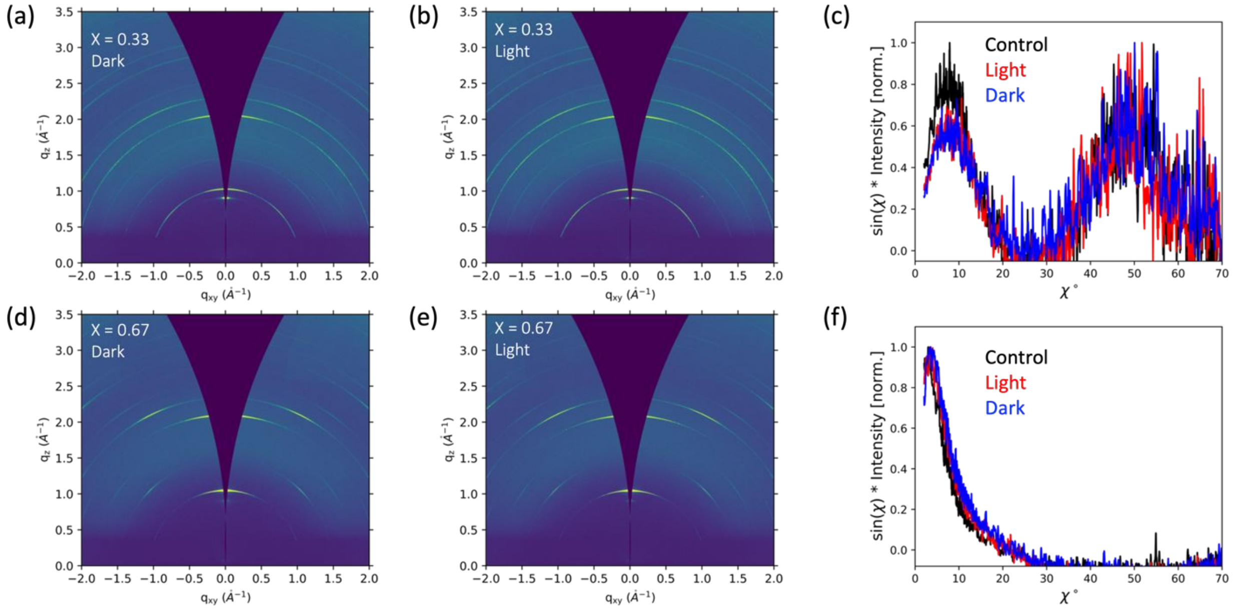

Figure S2: Effect of aging environment on orientation of $MAPbX_3$ films as seen in: (a-b) 2D GIWAXS images for $MAPbI_2Br$ and (c) associated pole figure for samples aged in the dark (blue), in the light (red), and in a dry inert environment (black); and (d-e) 2D GIWAXS images for $MAPbIBr_2$ and (f) associated pole figure for samples aged in the dark (blue), in the light (red), and in a dry inert environment (black).

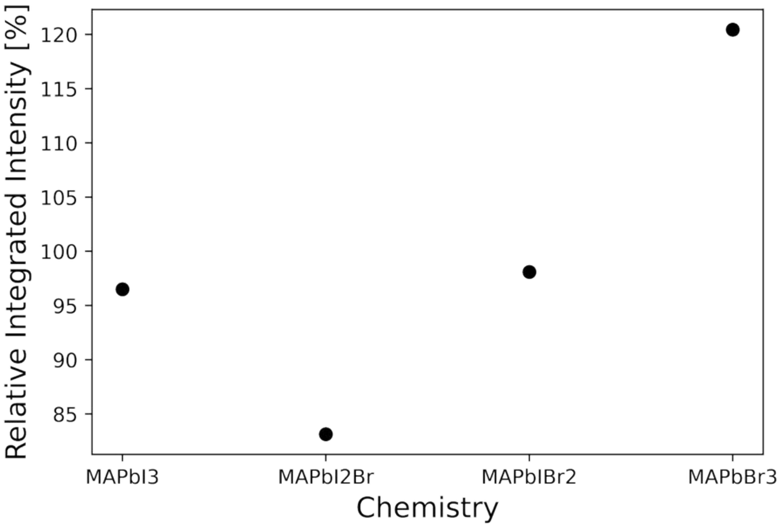

Figure S3: Effect of light-soaking on integrated intensity of the perovskite pseudocubic [100] diffraction peak. Integrated intensity is light aged sample relative to dark aged sample. Samples from the same chemistry are from the same original film so differences in thickness and orientation are expected to be minimal.

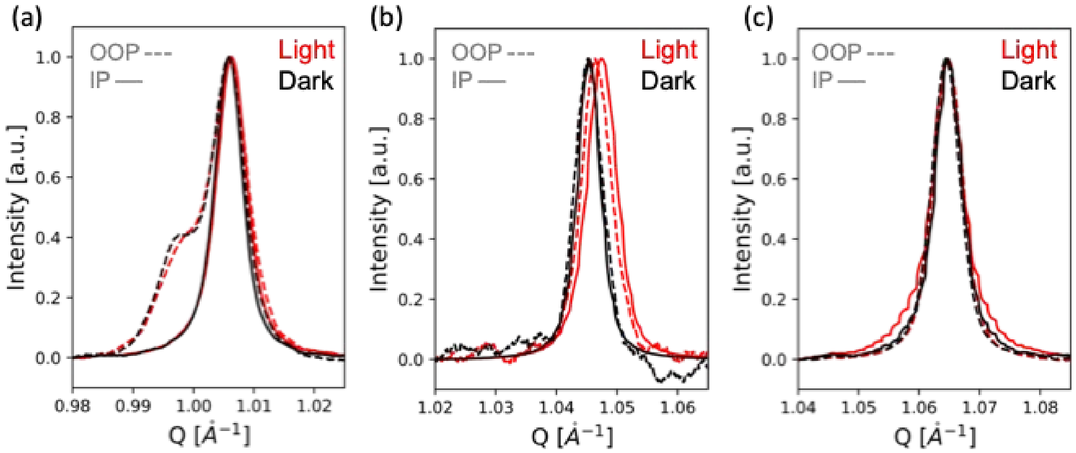

Figure S4: Normalized in-plane (IP) versus out of plane (OOP) diffraction for (a) $MAPbI_3$ (b) $MAPbIBr_2$ and (c) $MAPbBr_3$ for films aged in either the light (red) or dark (black) in toluene.

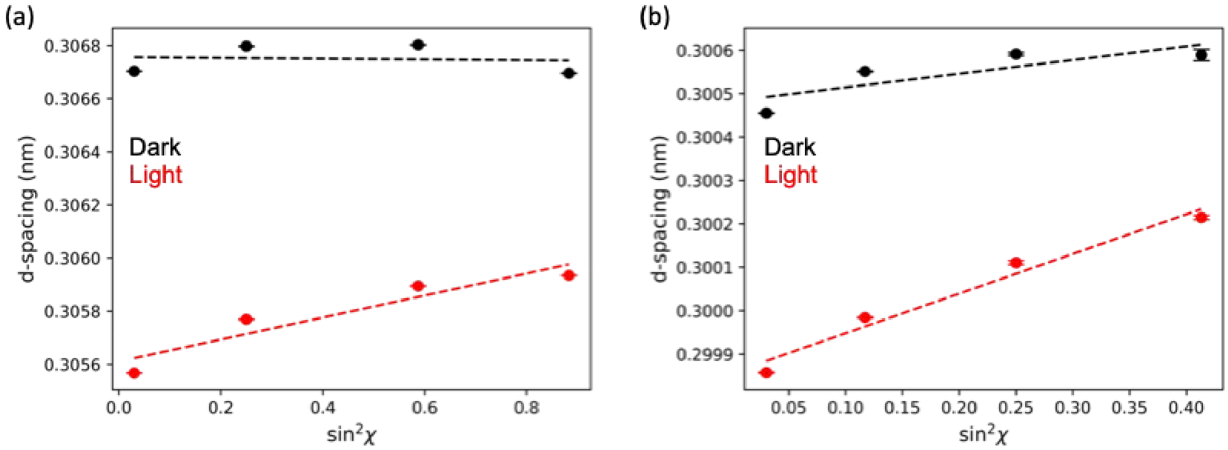

Figure S5: Effect of light-soaking on residual strain in (a) $MAPbI_2Br$ and (b) $MAPbIBr_2$ perovskite thin films aged in toluene. D-spacing of the [200] perovskite peak and associated uncertainty determined from pseudo-Voigt fitting. The slope of the line of best fit (dashed line) is proportional to in-plane residual stress.

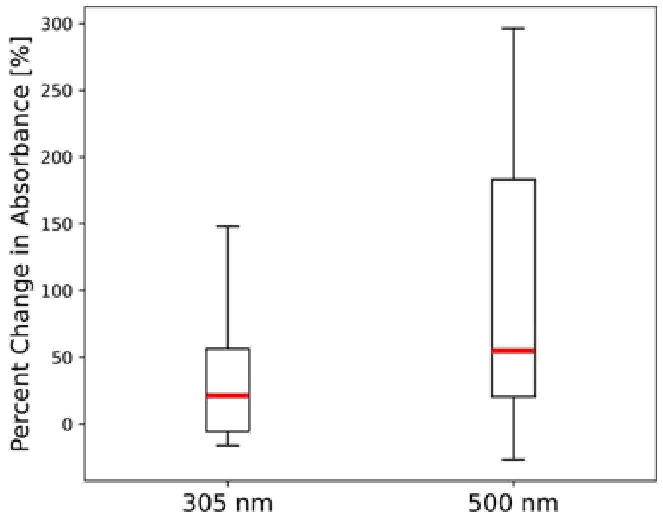

Figure S6: Percent change in absorbance of toluene solutions as function of perovskite light soaking. Comparison is between samples stored in the dark and in the light for 24-hours. Box plots represent data from 16 experiments using iodine containing perovskites.

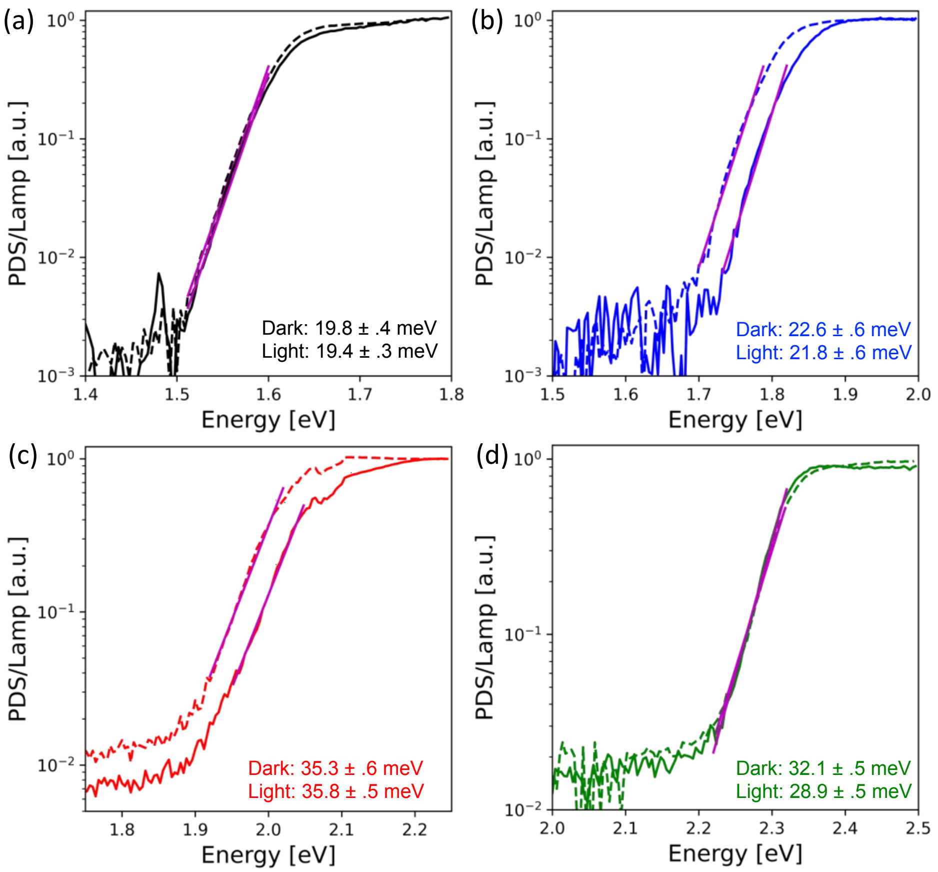

Figure S7: Determination of Urbach energy from PDS data for perovskite samples aged in toluene in the dark (dashed line) and light (solid line) for $MAPbI_3$ (a), $MAPbI_2Br$ (b), $MAPbIBr_2$ (c), and $MAPbBr_3$ (d). Line of best fit shown in magenta and determined Urbach energies included in inset.

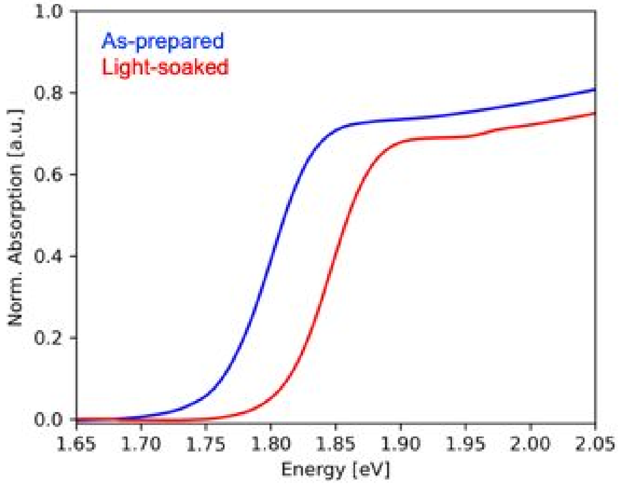

Figure S8: Effect of aging on the absorption of an encapsulated $MAPbBrI_2$ film. Absorption of a film as-prepared (blue) and after (red) 48-hours of light soaking under 1-sun,

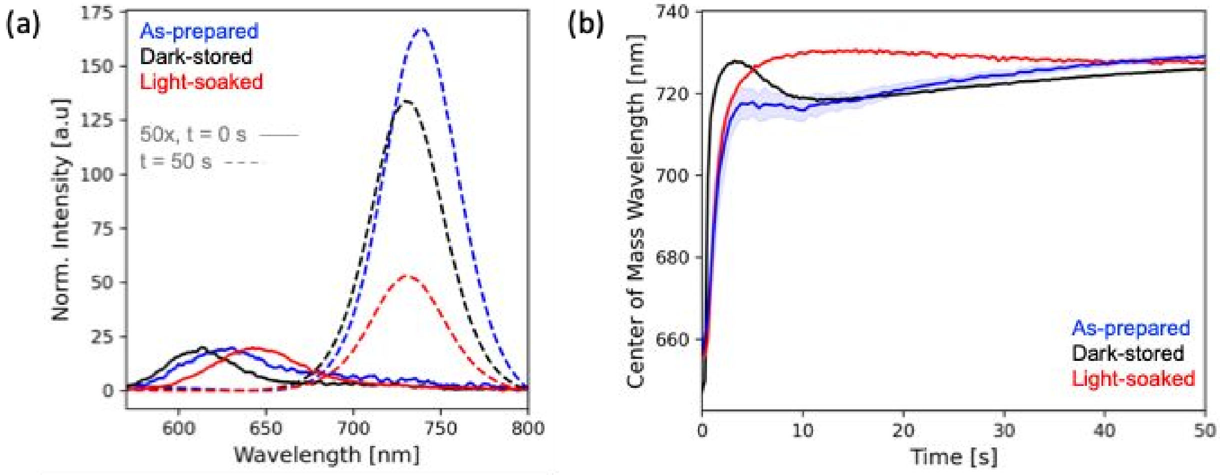

Figure S9: Effect of aging on $MAPbIBr_2$ films on photoluminescence. (a) Impact on photoluminescence spectra comparing films as-prepared (blue), after dark-storage, and after light-soaking under 1-sun illumination for 48 hours. Initial spectra (solid lines) and spectra after 7.5 minutes of ~3-suns equivalent of 450 nm CW laser illumination. (b) Evolution of the intensity-weighted mean photoluminescence wavelength (center of mass wavelength) with ~ 3-suns equivalent of 450 nm CW laser illumination for samples before aging (blue) stored in the dark for 48 hours (black) and stored under 1-sun for 48 hours (red). Shaded blue region shows range of response for unaged samples.

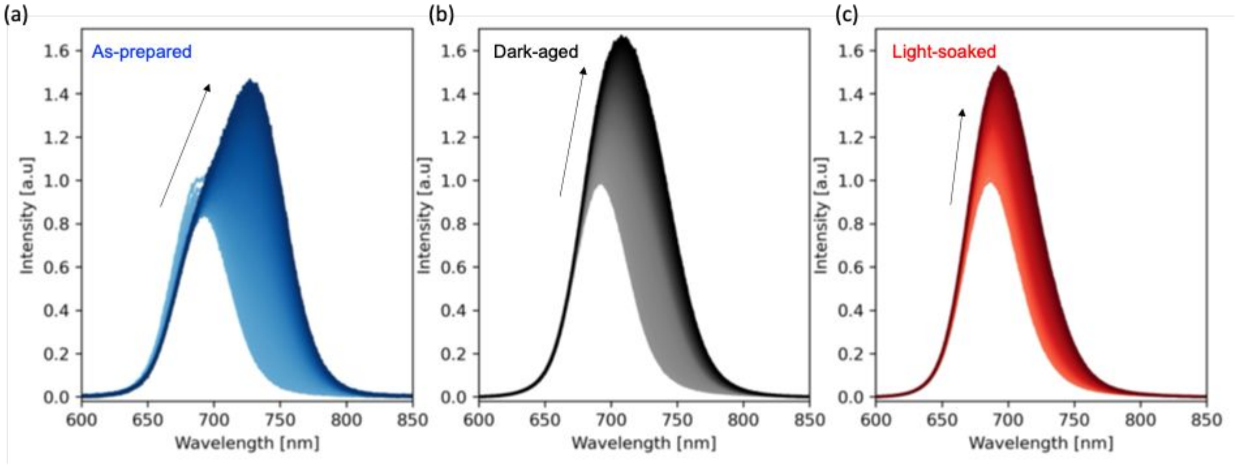

Figure S10: Evolution of PL spectra under ~3-suns equivalent of 450 nm CW laser illumination showing emission from an (a) unaged, (b) stored in the dark, and (c) stored under 1-sun illumination. Direction of PL evolution overtime highlighted with arrow.

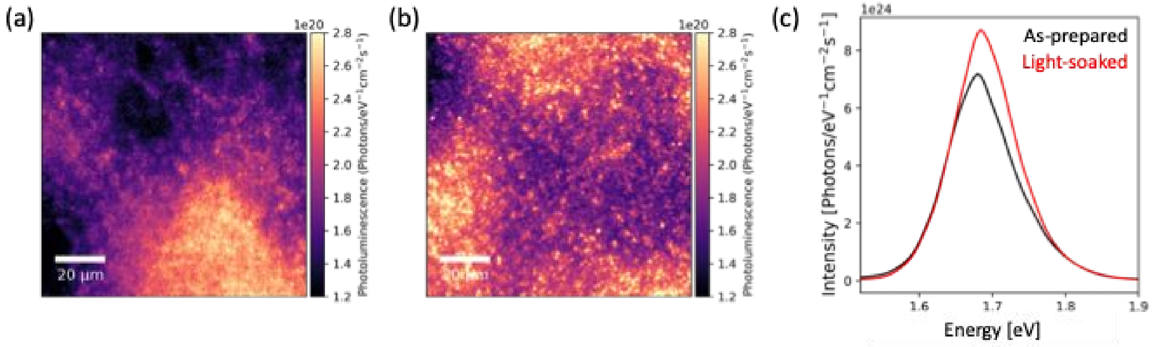

Figure S11: Impact of light soaking on photoluminescence intensity. Maps of absolute photoluminescence intensity of $MAPbI_2Br$ film as-prepared (a) and after (c) 48 hours of light-soaking under 1 sun. Comparison of integrated PL spectra for mapped areas for fresh (black) versus aged (red) $MAPbI_2Br$ film.

SI Note 1

Estimate of Perovskite Composition

To estimate changes in the perovskite composition as a function of aging, Vegard's law and an experimentally determined bandgap model were used for approximation with XRD and PDS data respectively. In the case of XRD data, the prior experimentally determined relationship of $< a > = 6.276\text{Å} - 0.37\text{Å}\, x$ (where x is bromine fraction) was used.[1] Noting a lattice contraction of ~0.02 Å for $MAPbI_2Br$ and ~0.01 Å for $MAPbIBr_2$, we estimate the absolute iodine increase to be approximately 3-5% with light exposure. For the PDS data, the previously published model of $E_g(x) = 1.57\text{eV} + 0.39\text{eV}x + 0.33\text{eV}x^2$ (where x is bromine fraction) was used to estimate bandgap changes.[2] In this case, observing a bandgap increase of ~0.36 eV for $MAPbI_2Br$ and ~0.24 eV for $MAPbIBr_2$, we estimate the absolute iodine increase in these samples to be 3-4%.

SI Note 2

Potential Products of $APbX_3$ Photolysis

Under illumination several light-driven reactions in lead-halide perovskites have been proposed. In the case of mixed-halide perovskites, these include proposed reactions that result in the full or partial dissolution of a perovskite phase, and those that drive the formation of an increasingly defective perovskite lattice. Adapted reaction schema for perovskites under illumination from P. Mathew, et al.[3] and Z. Xu, et al.[4] are shown below:

$$2MAPb(I_{0.5}Br_{0.5})_3 \rightarrow MAPbBr_3 + PbI_2 + MA^+ + I^- \quad (1)^{[3]}$$
$$4CsPb(I_{0.5}Br_{0.5})_3 \rightarrow 4CsPbBr_{1.5} + 3I_2 + 6V_I^+ + 6e^- \quad (2)^{[4]}$$

In both cases these reactions should be seen as possible steps in a degradation process, not necessarily the end state for a given perovskite film. The reaction described in eq. 1 represents a decomposition pathway that produces PbI_2 and ionic species. In contrast, the reaction described in eq. 2 represents a defect-formation reaction driven by iodide oxidation. Here, $CsPbBr_{1.5}$ is used to describe the bromide-containing perovskite after iodine removal. In the case of reactions of the style presented in eq. 2, the impact of the excess electron population and the ability to maintain a highly -defective perovskite lattice without subsequent dissolution remain open to discussion. While previous work has shown evidence of increases in electron-concentration with photolysis (suggesting a maintained electron population as hypothesized in equation 2),[4–6] we cannot rule out alternative reduction products such as the formation of reduced lead species including metallic Pb^0 at this time. Future work should look to clarify these products, particularly as they pertain to formation under device-relevant experimental conditions.

SI Note 3

Estimate of Iodine Loss into Solution with Light

To estimate iodine loss from the perovskite phase into solution as a function of light-soaking an approximation was made using Beer-Lambert's Law. The concentration of iodine in toluene was estimated using the published extinction coefficient of I_2 in toluene and the measured peak absorbance at ~500 nm.[7] From those measurements we estimate an increase in I_2 of 1 µM for the $MAPbI_3$ sample and 6-7 µM for the $MAPbBrI_2$ and $MAPbBr_2I$ samples. Using the geometry of the sample (~1 cm^2 and ~300 nm thick film) and the volume of toluene in the experiment (1 mL) we compare the approximate original moles of iodine in the perovskite to the measured iodine in solution. In doing so we approximate an absolute iodine loss of ~2 % for the mixed-halide samples.

[1] G. C. Halford, Q. Deng, A. Gomez, T. Green, J. M. Mankoff, R. A. Belisle, *ACS Appl. Mater. Interfaces* **2022**, *14*, 4335.

[2] J. H. Noh, S. H. Im, J. H. Heo, T. N. Mandal, S. Il Seok, *Nano Lett.* **2013**, *13*, 1764.

[3] P. S. Mathew, G. F. Samu, C. Janáky, P. V. Kamat, *ACS Energy Lett.* **2020**, *5*, 1872.

[4] Z. Xu, X. Zhong, T. Hu, J. Hu, A. Kahn, B. P. Rand, *J. Am. Chem. Soc.* **2024**, *146*, 33368.

[5] M. Lee, J. A. Vigil, Z. Jiang, H. I. Karunadasa, *Chem. Sci.* **2025**, *16*, 9662.

[6] J. A. Vigil, N. R. Wolf, A. H. Slavney, R. Matheu, A. Saldivar Valdes, A. Breidenbach, Y. S. Lee, H. I. Karunadasa, *ACS Cent. Sci.* **2024**, DOI 10.1021/acscentsci.4c00056.

[7] H. A. Benesi, J. H. Hildebrand, *J. Am. Chem. Soc.* **1949**, *71*, 2703.
